# Photoresponse Dimensionality of Organic Field-Effect Transistor

**DOI:** 10.3390/ma14237465

**Published:** 2021-12-06

**Authors:** Tomas Vincze, Michal Micjan, Juraj Nevrela, Martin Donoval, Martin Weis

**Affiliations:** Institute of Electronics and Photonics, Slovak University of Technology in Bratislava, Ilkovicova 3, 81219 Bratislava, Slovakia; tomas.vincze@stuba.sk (T.V.); michal.micjan@stuba.sk (M.M.); juraj.nevrela@stuba.sk (J.N.); martin.donoval@stuba.sk (M.D.)

**Keywords:** organic field-effect transistor, photoresponse, organic semiconductors

## Abstract

Organic field-effect transistors have been envisioned for advanced photodetectors because the organic semiconductors provide unique absorption characteristics, low-cost fabrication, or compatibility with flexible substrates. However, the response time of organic phototransistors still does not reach the required application level. Here, we report the photoresponse of copper phthalocyanine phototransistor in a steady state and under pulsed illumination. The detailed analysis based on the random walk among a field of traps was used to evaluate the dimensionality of electron transport in a device.

## 1. Introduction

The 21st century is denoted as a photonic age since the generation, transfer, modulation, and detection of photons is in the focus of science and technology. Since the signal processing is done mainly by electronics, the photogeneration of charges in electronic devices is crucial for converting light into an electric signal. Most commercial photodetectors are used on inorganic semiconductor-based photodiodes because of the high mobility of free carriers, high stability, and low exciton binding energy [1,2]. The significant drawbacks are the production costs, limiting large-area fabrication, or spectral properties, reducing application possibilities.

Organic semiconductors are organic materials with electronic properties similar to inorganic semiconductors. The unique optical properties of organic semiconductors originating from the energy band of molecular materials or low-cost deposition technology make them an alternative to inorganic semiconductors for optoelectronic devices [2,3,4,5]. Although these materials are denoted as semiconductors, organic semiconductors possess differences from traditional inorganic semiconductors. The doping level does not control the conductivity of organic semiconductors; however, the conductivity is defined by the intrinsic nature of the charge transport [6]. In other words, the organic semiconductor with *p*-type conductivity represents a material with effective mobility of holes (major carriers) much higher than the electrons (minority carriers).

Even though the organic photodiodes are mostly used for photodetector fabrication [4], organic phototransistors have been envisioned to be used in various optoelectronic applications, such as a photodetectors and image sensors, optoelectronic switches, and memory devices [3]. The great advantage of organic field-effect transistor (OFET) devices is the signal gain in the device, while the drawback is the slower response (detection bandwidth). The response time of organic phototransistors is usually limited by the lifetime of the photogenerated minority carrier and not the comparatively short transit time of the majority carrier [7].

Here, we report the photoresponse of the OFET device with copper phthalocyanine as an organic semiconductor. The fundamental photodetector properties were evaluated to illustrate the photoconductive gain. Afterwards, the photoresponse under pulsed illumination was used to study the transient phenomena. The detailed analysis based on a random walk among a field of traps was used to evaluate the dimensionality of electron transport in the OFET device.

## 2. Materials and Methods

Organic field-effect transistors (OFETs) were fabricated using copper phthalocyanine (CuPc) as an active semiconductor layer. The heavily doped Si wafers served as a substrate and the gate electrode, while the thermally grown silicon dioxide layer was used as the gate insulator (110 nm). To improve the surface properties of gate insulator, the 30-nm-thick layer of poly(methyl methacrylate) (PMMA, supplied by Merck, Kenilworth, NJ, USA) was spun on the substrate prior to deposition of the organic semiconductor CuPc (triple-sublimed grade, >99.95% purity, supplied by Merck, Kenilworth, NJ, USA). The 100-nm-thick organic semiconductor layer was deposited by thermal evaporation in a high vacuum using the SPECTROS 100 deposition system (Kurt J. Lesker, Jefferson Hills, PA, USA) at a pressure lower than 10^−5^ Pa and a constant evaporation rate of 3 nm/min. Subsequently, the 60-nm-thick source and drain copper electrodes were deposited through the shadow mask that provided channel width W of 2.5 mm and channel length L of 50 µm. All of the film thicknesses were verified using the stylus profiler Dektak 150 (Bruker, MA, USA) and spectroscopic ellipsometer PHE 102 (Angstrom Advanced Inc, Stoughton, MA, USA). The electrical measurements were done in the ambient atmosphere using the semiconductor parametric analyzer B1500A (Keysight, CA, USA). The OFET device illumination was done using a white LED with a power density of 60 mW/cm^2^.

## 3. Results and Discussion

**Figure 1:**
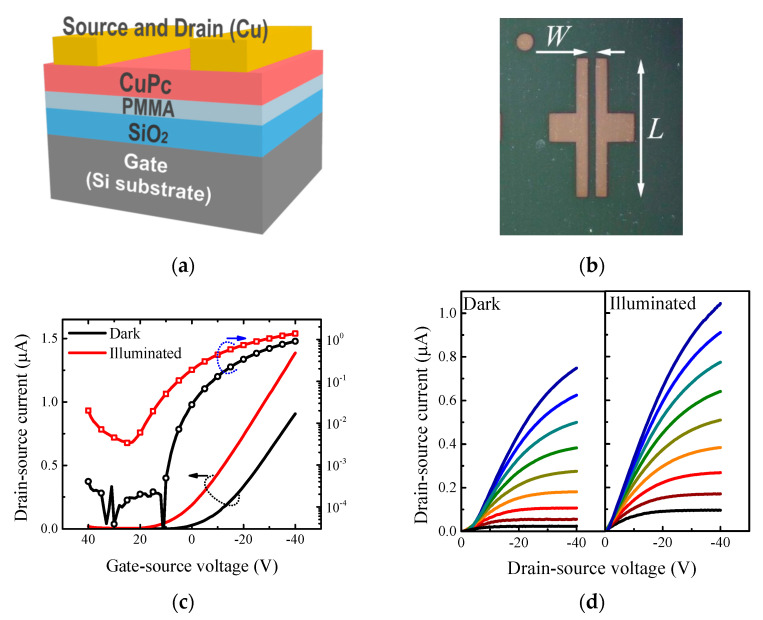
(**a**) Schematic view of the CuPc organic field-effect transistor and (**b**) the micrograph illustrating the electrode topology with channel width W and channel length L. (**c**) Transfer characteristics and (**d**) Output characteristics of CuPc OFET device in dark and under illumination. The transfer characteristics were carried out for a drain-source voltage of −40 V, whereas the output characteristics were recorded for a gate-source voltage that varied from 0 to −40 V (step 5 V).

To prove the hole-transport properties of CuPc, the OFET output and transfer characteristics were investigated. Figure 1 depicts the electrical characteristics of the OFET device. The threshold voltage $V_{th}$ and effective mobility μ were estimated by the gradual channel approximation model in the saturation region:

(1) $$I_{ds}=C_{g}\mu \frac{W}{L}{\left(V_{gs}-V_{th}\right)}^{2},$$

where $C_{g}$ is the capacitance per unit of area and $V_{gs}$ is the gate-source voltage. The OFET device exhibited common transistor behavior with effective mobility of about 10^−3^ cm^2^·V^−1^·s^−1^ and the threshold voltage of 9.3 V. The estimated value of effective mobility was in good agreement with the reported values 10^−4^–10^−3^ cm^2^·V^−1^·s^−1^ [8,9,10]. It should be mentioned here that the major part of the organic semiconductors suitable for organic field-effect transistors have very low photoresponse (e.g., pentacene), while organic semiconductors with high photoresponse are not suitable for organic field-effect transistor devices (e.g., poly(3-hexylthiophene-2,5-diyl)). Note that the CuPc OFET output current rose exponentially in the subthreshold region. The subthreshold slope $\left(\partial I_{ds}/\partial V_{gs}\right)$ for constant $V_{ds}$ expressed how abrupt the transition was from the off state to the on state. Notably, the subthreshold slope’s reciprocal value, the so-called subthreshold swing, is commonly used since it clearly describes the voltage required to increase the current by one decade (V/dec). The CuPc OFET device exhibited a sufficient ratio of output current in On and Off states of 10^4^ and the subthreshold swing of 8.6 V/dec; the high value was mainly caused by the higher thickness of the gate insulator layer. It should be noted here that the CuPc OFET properties were in close agreement with previously reported values. Obviously, the device illumination caused a significant rise in the drain-source current. Performing the same analysis as mentioned above, we estimated a slightly higher mobility of 1.5 × 10^−3^ cm^2^·V^−1^·s^−1^ and a drastically shifted threshold voltage of 25.8 V. The positive shift of the threshold voltage represented a generation of the negative charge with a surface density of 2.3 × 10^12^ e/cm^2^ (estimated as $Q=∆V_{th}C_{g}$, where $∆V_{th}$ represents the threshold voltage shift). The presence of additional charges also suppressed the On/Off ratio to the value of 10^3^ and increased the subthreshold swing to 10.7 V/dec. In other words, the OFET device exhibited charges even in the closed channel due to the photogeneration. Interestingly, assuming the interface trap states’ density $N_{it}$ at the organic semiconductor–gate insulator interface, the subthreshold swing should follow the relation

(2) $$SS=\frac{kT}{e}ln\left(10\right)\left(1-\frac{eN_{it}}{C_{g}}\right),$$

where kT/e stands for the thermal voltage. The evaluated interface trap states’ density of 1.3 × 10^12^ cm^−2^ eV^−1^ was in good agreement with photogenerated charge surface density; hence, the photogenerated charges were trapped by localized states. Table 1 compares fundamental OFET parameters evaluated for the device in the dark and under illumination.

Since the CuPC OFET device exhibited photoresponse, the fundamental photodetector parameters were evaluated to illustrate the device performance. The responsivity R was given as a ratio of the device output current and the incident light power. The device exhibited responsivity of 30 mA·W^−1^ for the gate-source and drain-source voltages of −40 V. Since the responsivity was strongly dependent on the device dimensions, the photosensitivity P was more suitable for general description since it stood for the ratio of output currents of the device under illumination and in the dark. The CuPc OFET showed the photosensitivity of higher than 10^2^ for the gate-source voltage of 14 V, which confirmed the presence of photogenerated charges. Another crucial parameter used to evaluate the photodetector performance was the noise equivalent power, NEP. It was defined as the detection limit of the photodetector, and it follows the relation

(3) $$NEP=\frac{i_{n}}{R},$$

where $i_{n}$ is the total noise current in A·Hz^−1/2^. Assuming the shot noise from dark current $I_{d}\;$ is a dominant contribution that limits the photodetector, the total noise current is simplified into $i_{n}=\sqrt{2eI_{d}B},$ where e stands for the elementary charge and B is the bandwith typically assumed to be equal 1 Hz. As a result, the NEP reached the value of 12 nW·Hz^−1/2^. Finally, the specific detectivity $D^{*}$ illustrated signal-to-noise performance normalized by the photodetector area A as $D^{*}=A^{1/2}/NEP$ and it went up to the level of 9 × 10^10^ Jones. Table 2 summarizes the CuPc OFET photodetector parameters.

**Figure 2:**
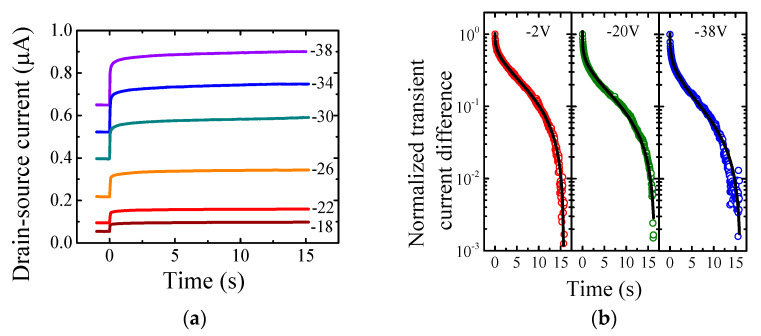
(**a**) The transient drain-source currents in time domain recorded for various gate-source voltages. The time 0 s denotes beginning of the illumination; hence, the current in negative time region represents the dark current. (**b**) The normalized transient current difference in semi-log scale for selected gate-source voltages. Solid lines represent the stretched exponential function fit.

Since the steady-state analysis above confirmed the photogeneration of charges in the channel region of the OFET device, the analysis of transient currents was applied to investigate the charge transport phenomenon. Photoswitching behavior under pulsed illumination was illustrated by the transition from the dark current to the light current, as shown in Figure 2a.

The transient phenomenon was investigated for gate-source voltages from 0 to −40 V. Interestingly, the recorded slow relaxation process did not follow a simple exponential function; however, the transient current I(t) obeyed the stretched exponential function, also recognized as Kohlrausch–Williams–Watts function:

(4) I(t)=Idark+∆I exp(−(tτ)β),

where $∆I=I_{light}-I_{dark}$ stands for the current difference, τ is the relaxation time, and β is the stretching exponent. Figure 2b depicts the suitability of the stretched exponential function for the normalized transient current difference. This nature has been reported for a wide variety of physical quantities in many different systems [11,12,13,14], including the carrier relaxation in semiconductors [15]. Unfortunately, most of the models of stretched exponential have no clear physical background since it can also represent a sum of exponential decays, i.e., the distribution of relaxation times [11]. On the other hand, the stretched relaxation can be applied for the hopping transport in disordered semiconductors due to its association with the presence of trap states [15,16,17]. Note that the most common charge transport model, both the Gaussian disorder model [18] and the multiple-trapping-and-release model [19], assume transport through the shallow traps of disordered semiconductors. Trap-limited dispersive transport can be described by the continuous-time random walk [20,21,22], and the stretching exponent β is closely related to the random walk dimensionality d as β=d/(d+2) [23].

**Figure 3:**
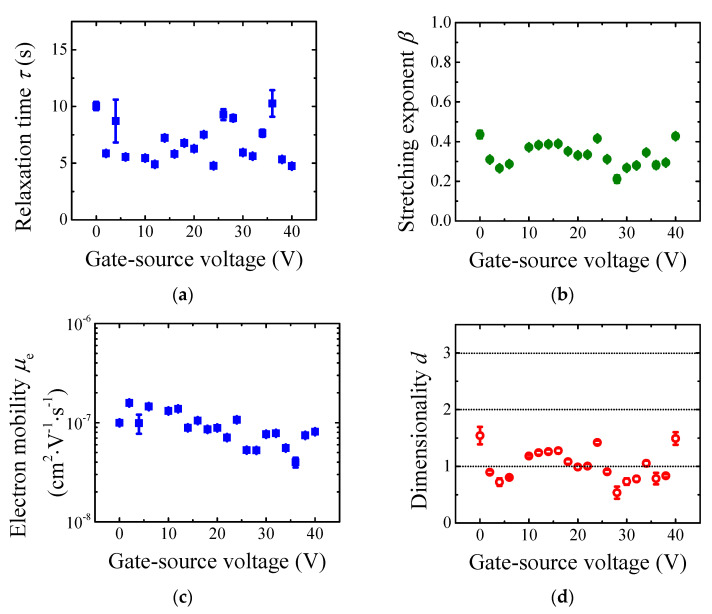
Fitting result of (**a**) relaxation time τ and (**b**) stretching exponent β as a function of the gate-source voltage. (**c**) Electron mobility $\mu_{e}\;$ evaluated from relaxation time and (**d**) charge transport dimensionality d evaluated from stretching exponent.

Figure 3a,b shows an estimated relaxation time τ and stretching exponent β as a function of the gate-source voltage. Interestingly, there is no significant voltage dependence of these parameters. The relaxation time τ can be used for an electron mobility evaluation. Assuming the time-of-flight model for OFET device as a first approximation [24,25],

(5) μe≃L2τ|Vgs−Vth|,

We achieved electron mobility in the order of 10^−7^ cm^2^·V^−1^·s^−1^, as shown in Figure 3c. Noteworthy, the electron traps caused extremely low electron effective mobility in CuPc film. As stated above, the stretching exponent β represents the charge transport dimensionality d. Figure 3d depicts the electron transport dimensionality d. Obviously, the channel region discharging was a one-dimensional process that agreed with electron photogeneration across the bulk of the organic semiconductor [26]. In other words, the electron transport was not only on the organic semiconductor/gate insulator interface as described by gradual channel approximation [27]; however, the electrons were present in the bulk of the organic film. It should be noted that electron hopping discussed here was caused by high density of trap states (i.e., multiple-trapping-and-release model can be applied) and it was not an intrinsic feature of electron transport in CuPc films. Furthermore, even though the detailed analysis was illustrated on a single device, the photogenerated charge transient phenomenon was in the same manner observed on the whole family of fabricated OFET devices (56 in total).

## 4. Conclusions

The photoresponse of CuPC OFET device was investigated in steady state and under pulsed illumination. The steady-state parameters verified its suitability as a photodetector, while the pulsed illumination was applied to study the transient phenomena. The detailed analysis based on the random walk among a field of traps was used to evaluate the transport of electrons as minority charge carriers. The observation of stretched exponential behavior of a transient nature confirmed the random walk of electrons between traps. The relaxation time provided electron mobility on the level of 10^−7^ cm^2^·V^−1^·s^−1^, and the stretching exponent evaluation showed the one-dimensional nature of the electron transport. As a result, we can conclude that the roadmap of the organic phototransistor development must include enhancing minority charge carriers’ mobility by suppressing trap density.

## Figures and Tables

**Table 1:** CuPc OFET device parameters.

Parameter	Value	
	Dark	Illuminated
Effective mobility (×10^−3^ cm^2^·V^−1^·s^−1^)	1.06 ± 0.01	1.50 ± 0.01
Threshold voltage (V)	9.3 ± 0.1	25.8 ± 0.1
Subthreshold swing (V/dec)	8.6 ± 0.1	10.7 ± 0.1
On/Off ratio	10^4^	10^3^

**Table 2:** Photodetector parameters evaluated for the CuPc OFET device.

Parameter	Value
Responsivity *R* (mA·W^−1^)	30
Photosensitivity *P*	104
Noise equivalent power *NEP* (nW·Hz^−1/2^)	12
Detectivity D* (Jones)	9 × 10^10^

## Data Availability

The data presented in this study are available in article and Supplementary Material.

## Supplementary Materials

The following are available online at https://www.mdpi.com/article/10.3390/ma14237465/s1.
